# Cell-culture model to study endothelial activation in sepsis

**DOI:** 10.1186/cc14119

**Published:** 2015-03-16

**Authors:** T Eichhorn, S Rauscher, C Hammer, B Führer, M Gröger, V Weber

**Affiliations:** 1 Danube University Krems, Austria; 2 Core Facility Imaging, Medical University of Vienna, Austria

## Introduction

The endothelium is a complex organ influenced by circulating mediators, adjacent cells, physico-chemical factors, and shear stress. During systemic inflammation and sepsis, excessive and sustained activation of the endothelium result in the loss of its anticoagulant and anti-adhesive characteristics as well as in a loss of endothelial barrier function. We set up a cell-culture model to study endothelial activation induced by lipopolysaccharide (LPS) or by plasma from septic patients and studied the effect of adsorbent-based mediator modulation on endothelial activation.

## Methods

Human whole blood was stimulated with LPS (100 ng/ ml) from *Escherichia coli *for 4 hours. The stimulated blood or plasma from septic patients was treated *in vitro *with 10 vol% polystyrene- divinylbenzene (PS-DVB)-based polymers (CG161, mean pore size 16 nm; CG300, mean pore size 30 nm) or left untreated. After adsorption, the plasma was separated and diluted with cell culture medium. The resulting conditioned medium was used to stimulate human umbilical vein endothelial cells (HUVEC) for 16 hours. HUVEC activation was assessed by the release of interleukin (IL)-1β, IL-6, IL-8, IL-10, and tumor necrosis factor (TNF)-α, plasminogen activator inhibitor-1 (PAI-1), as well as the expression of intercellular adhesion molecule (ICAM)1 and E-selectin. HUVEC were cultured at a shear stress of 5 dyne/ cm^2 ^using the Ibidi perfusion system. Adhesion of monocytic THP-1 cells to HUVEC was studied after 4 hours of HUVEC stimulation with conditioned media. THP-1 cells were perfused over HUVEC at 1 dyne/ cm^2 ^for 15 minutes, and adhering THP-1 were quantified over time.

## Results

The adsorbents CG161 and CG300 substantially decreased levels of TNFα, IL-1β, IL-6, IL-8 and IL-10 in LPS-stimulated blood. TNFα, a key stimulus for HUVEC, was reduced to 12% and 8% of the initial concentration by CG161 and CG300, respectively. Stimulation of HUVEC with the adsorbent-treated plasma resulted in significantly diminished release of IL-6, IL-8, PAI-1 and decreased ICAM-1 and E-selectin expression, indicating reduced HUVEC activation. THP-1 adhesion was substantially decreased when HUVEC were stimulated with CG300-treated plasma as compared with untreated controls.

## Conclusion

The flow model allows to study the effect of cytokine modulation on endothelial activation and to assess the interaction of activated endothelial cells with blood cells. Modulation of inflammatory mediators with porous polystyrene-based polymers attenuates endothelial activation and reduces monocyte adhesion.

